# Evaluation of Effect of Dietary Supplementation with Microencapsulated Hydrolyzed Tannins on Growth, Slaughter Performance, Meat Quality, and Lipid Metabolism of Zhongshan Shelducks

**DOI:** 10.3390/foods14050839

**Published:** 2025-02-28

**Authors:** Zhimei Tian, Zhengwei Tian, Yingshan Yin, Yongmei Wu, Zhenyuan Li, Qiaohua Liang, Miao Yu, Yiyan Cui, Xianyong Ma, Guanghui Peng, Zhenming Li

**Affiliations:** 1 State Key Laboratory of Swine and Poultry Breeding Industry, Key Laboratory of Animal Nutrition and Feed Science in South China, Ministry of Agriculture and Rural Affairs, Guangdong Provincial Key Laboratory of Animal Breeding and Nutrition, Institute of Animal Science, Guangdong Academy of Agricultural Sciences, Guangzhou 510640, China; tianzhimei@gdaas.cn (Z.T.); yumiao@gdaas.cn (M.Y.); cuiyiyan@gdaas.cn (Y.C.); maxianyong@gdaas.cn (X.M.); pengguanghui@gdaas.cn (G.P.); 2 Zhongshan Agricultural Science and Technology Extension Center, Zhongshan 528400, China; jc_12323@163.com (Y.Y.); wuyongmeizi@163.com (Y.W.); LZY1983ZY@163.com (Z.L.); 121014918@qq.com (Q.L.)

**Keywords:** microencapsulated hydrolyzed tannins, growth, meat quality, lipid metabolism, productivity, Zhongshan shelducks

## Abstract

This study investigated the effects of microenapsulated hydrolyzed tannins (MHTs) on the growth performance and meat quality of Zhongshan shelducks. A total of 288 healthy Zhongshan shelducks with an average initial weight of 1790.27 ± 0.14 kg were randomly divided into four groups through a 56 d experiment period and were fed a basal diet supplemented with 0 (CON), 400, 800, and 1600 mg/kg MHTs, respectively. Results showed that 400 and 800 mg/kg MHTs improved the final body weight, average daily gain, glutathione peroxidase activity, and total antioxidant capacity compared to CON (*p* ≤ 0.05). The diet supplemented with 400 mg/kg MHTs decreased shear force and 800 mg/kg MHTs increased the yield of *pectoralis major* muscle compared to CON (*p* ≤ 0.05). Dietary MHTs increased inosine monophosphate content and decreased percentage C14:0 content in meat; however, the b*45 min value, 48 h drip loss, and shear force were increased but the percentage intramuscular fat (IMF) content was decreased in *pectoralis major* muscle with the increase in MHTs (*p* ≤ 0.05). Compared to CON, 400 and 800 mg/kg MHTs increased the percentage content of IMF, C18:1n-9, C18:2n-6, monounsaturated fatty acids, polyunsaturated fatty acids, and unsaturated fatty acids in *pectoralis major* muscle (*p* ≤ 0.05). Furthermore, 400 and 800 mg/kg MHTs improved the lipid metabolism of IMF deposition, fatty acid uptake, and adipogenesis by activating the *peroxisome proliferator-activated receptor gamma* pathway to regulate *fatty acid synthetase* and *lipoprotein lipase* genes. In conclusion, diets supplemented with 400 and 800 mg/kg MHTs could improve growth, meat quality, antioxidant capacity, and lipid metabolism in Zhongshan shelducks.

## 1. Introduction

With the current demographic growth, production units need to improve their productive yield within the same territorial limits without increasing the number of specimens but also preserving the quality of the meat [1]. Tannins is a general term for a class of polyphenolic natural substances and they are classified into hydrolyzed tannins and condensed tannins based on their structures [2]. Hydrolyzed tannins are D-glucose-centered phenolic acid polyesters that can be decomposed into polyols and phenolic acids including gallic acid and trihydroxybenzoic acid [3,4]. Hydrolyzed tannins display antimicrobial, anti-inflammatory, antioxidant, and astringent properties as well as anticarcinogenic and antimutagenic potentials in animals, which are used as feed additives in livestock and poultry production [5,6,7]. Li et al. [8] indicated that 400 mg/kg hydrolyzed tannins promoted the feed conversion rate, digestion, absorption, and microbial flora of the intestine, as well as reducing the death and culling rate, of broilers. However, Buyse et al. [9] found that 500 mg/kg tannins had no effect but 2000 mg/kg tannins had a negative effect on the growth performance of broilers. Liu et al. [10] found that 1000 mg/kg hydrolyzed tannins did not influence growth but improved antioxidant capacity and intestinal morphology in weaned piglets. Therefore, these different results can probably be attributed to different animal species and antinutritive properties such as poor taste, feed intake, and nutrient digestibility in high-dose tannins.

Feed additives are microencapsulated to increase their preservation time and action time through controllable target site release and to improve their efficiency [11]. Xu et al. [2] showed that dietary supplementation with 1500 mg/kg microencapsulated tannins (extracted from chestnut and coated with hydrogenated palm oil) significantly improved the growth performance and intestinal digestive function of weaned piglets and reduced the mortality ratio. Wang et al. [12] reported that 500–1500 mg/kg microencapsulated tannins (extracted from Chinese gallnut) had no negative effect on growth performance; furthermore, 1000 mg/kg microencapsulated tannins improved intestinal morphology, intestinal nutrient transport, and the intestinal microbiota in weaned piglets. These inconsistent results may be attributed to different concentrations and sources of microencapsulated tannin supplementation. Moreover, Buyse et al. [9] found that 500 mg/kg chestnut tannins had a positive effect on meat quality such as the pH value, drip loss, and shear force in broilers. However, the effects and applicative doses of tannins on meat quality in shelducks remain unclear.

Zhongshan shelducks, as a dual-use breed, are a unique germplasm resource due to the culmination of a distinctive set of physical features and unique characteristics and are primarily distributed in Zhongshan city, Guangdong Province, China [13]. Until now, there have been few studies on tannins in Zhongshan shelducks, and little information is available about the effects of tannins on Zhongshan shelducks. One previous study found that diets supplemented with 1500 and 2500 mg/kg quebracho tannin did not affect the growth of slow-growing Muscovy ducks [14], while King et al. [15] reported that high-tannin diets inhibit nutrient utilization in White Pekin duck. Therefore, we infer that appropriate supplementation with microencapsulated tannins might promote the growth performance and improve the meat quality of Zhongshan shelduck. Growth performance, slaughter performance, and meat quality are the most direct indicators of the nutritional status, productivity, and economic value of livestock and poultry [1,16]. Antioxidant capacity can reflect the growth, health status, and meat quality of animals [1,17,18]. FAs are important flavor and nutrient substances within muscles and are closely related to meat quality and lipid metabolism such as lipid synthesis, lipolysis, intramuscular fat (IMF) deposition [19,20,21]. Therefore, it is crucial to regulate growth, productivity, antioxidant capacity, and lipid metabolism to maintain good health and meat quality in Zhongshan shelducks, which remain unclear and need to be investigated further.

The objective of the present study was to evaluate applicative doses and the effects of microencapsulated hydrolyzed tannins (MHTs) on growth performance, slaughter performance, meat quality, and lipid metabolism in Zhongshan shelduck. In this study, we hypothesized that diets supplemented MHTs may promote growth and meat quality by improving antioxidant capacity and lipid metabolism.

## 2. Materials and Methods

The experiment was conducted in accordance with the guidelines of animal welfare and approved by the Animal Care and Use Committee of Guangdong Academy of Agricultural Sciences (No. GAASIAS-2021-11).

### 2.1. Chemicals

MHTs (50% effective compositions of hydrolysable tannins that are D-glucose-centered phenolic acid polyesters composed of polyol and gallic acids (gallotannins) or ellagic acids (ellagitannins)) were obtained from Vétoquinol Ascor Pharmaceutical Group Co., Ltd. (Shanghai, China), which were extracted from chestnut and coated using cryogenic spray condensation coating technology.

### 2.2. Experiment Design and Animal Experiment

A single-factor, completely random test was conducted in this experiment. A total of 288 healthy (monitored according to China national standard [22]) Zhongshan shelducks of 12 weeks old with similar body weights (1790.27 ± 0.14 kg) were randomly divided into 4 dietary treatments with 6 replicative cages in each treatment with 12 ducks per cage. The ducks were fed a basal diet supplemented with 0 (CON), 400, 800, and 1600 mg/kg MHTs in 4 groups, respectively. The dosages of MHTs were supplemented in diets according to previous reports [2,12,14,23] and manufacturer-recommended doses. The basal diet was designed to meet nutrient requirements for ducks and is shown in Table 1. All ducks were caged by net-raising in a pond (5.00 by 2.50 m, 12.5 m^2^ of net surface). The ducks were fed with the restriction of feeding twice every day at 8:00 and at 18:00 through the 56 d experiment period, and the average daily intake per duck was about 209 ± 0.13 g. The initial body weight (IBW) and final body weight (FBW) after fasting for 12 h were recorded to calculate the average daily gain (ADG).

### 2.3. Carcass Traits Analysis and Sample Collection

After fasting for 12 h, 2 ducks per cage were randomly selected and slaughtered following electrical stunning and exsanguination. Postmortem, the ducks were defeathered, and the feet, head, internal organs, *pectoralis major* muscle, and bone-free left thigh muscle were dissected to calculate carcass traits like carcass yield, eviscerated yield, semi-eviscerated yield, *pectoralis major* muscle yield, and thigh muscle yield according to the China Industrial Standard [24]. The *pectoralis major* muscle samples of 1 duck per treatment were immediately collected and frozen in liquid nitrogen for the later determination of gene expression, antioxidant indexes, fatty acids, IMF, and inosine monophosphate (IMP). The *pectoralis major* muscle samples of another duck per treatment were used to detect meat quality.

### 2.4. Meat Quality Assay

Dissected *pectoralis major* muscle was used to determine meat quality, considering pH, meat color, drip loss, and shear force. At 45 min, 24 h, and 48 h postmortem, the pH and meat color CIE LAB (lightness (L*), redness (a*), and yellowness (b*)) values of *pectoralis major* muscle were measured using a pH meter (Testo-205, Testo, Lenzkirch, Germany) and colorimeter (CR-410, Minolta, Chiyoda, Japan), respectively. The drip loss and shear force were detected as described previously [25]. The cores (3 cm × 1 cm × 1 cm) of *pectoralis major* muscle after weighing were placed in sealed plastic bags and refrigerated at 4 °C for 24 h and 48 h and then were re-weighed after removing surface moisture using filter paper for calculating drip loss. The *pectoralis major* muscle refrigerated at 4 °C for 24 h was cooked to an internal temperature of 80 °C in a 90 °C water bath and then cooled to room temperature. The cylindrical cores (10 mm diameter × 10 mm length) of the cooked muscle were cut perpendicular to fiber orientation using an Instron machine (Instron model 4411, Instron Corp., Canton, MA, USA). Values were recorded to calculate shear force in Newtons (N).

### 2.5. IMF, IMP, and Fatty Acids of Pectoralis Major Muscle

IMP was detected using high-performance liquid chromatography as in a previous study [26]. IMF was extracted and analyzed using the Soxhlet system (Soxtec 2055, Foss Tecator AB, Foss, Sweden FOSS Analytical Instruments, Hilleroed, Denmark). Fatty acid composition was detected using gas chromatography analysis (Agilent6890, Agilent, Santa Clara, CA, USA) according the procedures of AOAC [27] and a previous study [25].

### 2.6. Muscular Antioxidant Indexes

The *pectoralis major* muscle was homogenized using 0.9% saline and centrifuged at 4 °C and 4000× *g* r/min for 20 min. The supernatant was collected for determining the total antioxidant capacity (T-AOC), total superoxide dismutase (T-SOD) activity, glutathione peroxidase (GSH-Px) activity, and malondialdehyde (MDA) content according to the manufacturer’s protocol for the kits (AO15-2-1, A001-1-2, A005-1-2, and A003-1-2, Nanjing Jiancheng Bioengineering Institute, Nanjing, China).

### 2.7. Quantitative Real-Time PCR (qRT-PCR)

The RNA concentration was detected using a nucleic acid protein detector (Nanodrop 2000, Thermo Fisher Scientific, Waltham, MA, USA) after extracting total RNA from *pectoralis major* muscle using Trizol reagent (TaKaRa Biotechnology (Dalian) Co., Ltd., Dalian, China). About 1 μg of total RNA was reverse-transcribed into cDNA according to the PrimeScript TM RT reagent Kit (TaKaRa, Dalian, China). qPCR was performed using specific primers [16] (Table 2) and TB GreenTM Premix Ex TaqTM (Takara Biotechnology, Dalian, China) with the CFX96 Real-Time PCR Detection System (Bio-Rad Laboratories, Hercules, CA, USA), and the expressive abundance of genes was analyzed according to our previous description [25].

### 2.8. Statistics and Analysis

Data were analyzed using GraphPad Prism 9 (GraphPad Software, Inc. San Diego, CA, USA). Dietary treatment was considered a fixed effect, and individual ducks were the experimental units, except for the IBW, FBW, and ADG, which were evaluated using a pen as the experimental unit. Statistical significance was determined through one-way analysis of variance (ANOVA) followed by Tukey’s post hoc test. Data are shown as the mean ± SEM, and *p* ≤ 0.05 indicates significant difference.

## 3. Results

### 3.1. Growth and Slaughter Performance

Effects of MHTs on growth and slaughter performance in Zhongshan shelducks are shown in Table 3. Diets supplemented 400 and 800 mg/kg MHTs improved the FBW and ADG compared to CON (*p* ≤ 0.05). Diet supplemented with 800 mg/kg MHTs improved *pectoralis major* muscle yield compared to diets with 0, 400, and 1600 mg/kg MHTs (*p* ≤ 0.05). The FBW and ADG in the 1600 mg/kg MHT group were higher than those in the 800 mg/kg MHT group (*p* ≤ 0.05) and there were no significant differences compared to the CON and 400 mg/kg MHT groups (*p* > 0.05). No significant differences were observed in the dressing percentage, semi-eviscerated yield, eviscerated yield, and thigh muscle yield among the four groups (*p* > 0.05).

### 3.2. Antioxidative Status in Pectoralis Major Muscle

Effects of MHTs on antioxidant status are shown in Table 4. The activity of GSH-Px was higher in the 400 and 800 mg/kg MHT group compared to that in the CON group (*p* ≤ 0.05) and was not significantly different between the 1600 mg/kg MHT and other three groups (*p* > 0.05). The activities of T-SOD and MDA showed no significant differences among the four groups (*p* > 0.05). In the 400 and 800 mg/kg MHT groups, muscles displayed higher T-AOC compared to that in the CON and 1600 mg/kg MHT groups (*p* ≤ 0.05). GSH-Px and T-AOC showed no significant differences between the 400 and 800 mg/kg MHT groups (*p* > 0.05).

### 3.3. Meat Quality

Results for meat quality at 45 min, 24 h, and 48 h postmortem are shown in Table 5. Postmortem, the b* value was higher in the 800 and 1600 mg/kg MHT groups compared to that in the 400 mg/kg MHT group at 45 min (*p* ≤ 0.05). No significant differences were observed in pH (at 45 min, 24 h, and 48 h) and meat color except the b* value at 45 min among the four groups (*p* > 0.05). Diets supplemented with MHTs did not affect the drip loss at 24 h and 48 h compared to the control diet (*p* > 0.05), but drip loss at 48 h was increased in the 1600 mg/kg MHT group compared to that in the 400 and 800 mg/kg MHT groups (*p* ≤ 0.05). The content of IMF was increased in the 400 and 800 mg/kg MHT groups compared to that in the CON and 1600 mg/kg MHT groups (*p* ≤ 0.05). Diets supplemented with MHTs increased IMP content compared to the CON group (*p* ≤ 0.05).

### 3.4. Fatty Acid Profile and Lipid Metabolism

The fatty acid composition of *pectoralis major* muscle in Zhongshan shelducks is shown in Table 6. Compared to CON, diets supplemented MHTs decreased the content of C14:0 (*p* ≤ 0.05) but did not affect the content of C11:0, C16:0, C18:0, C20:1n-6, C18:3n-3, and saturated fatty acids (SFAs) (*p* > 0.05). Diets supplemented with 400 mg/kg MHTs decreased the C12:0 content but increased the C16:1n-7 content, while 1600 mg/kg MHTs decreased the content of C12:0 and C16:1n-7 compared to CON (*p* ≤ 0.05). Diets supplemented with 400 mg/kg and 800 mg/kg MHTs increased the content of C18:1n-9, C18:2n-6, monounsaturated fatty acids (MUFAs), polyunsaturated fatty acids (PUFAs), and unsaturated fatty acids (UFAs) compared to CON (*p* ≤ 0.05). Diets supplemented with 400 mg/kg decreased the C20:2n-6 content but increased the C20:4n-6 content compared to CON (*p* ≤ 0.05). Furthermore, diets supplemented with 800 mg/kg and 1600 mg/kg MHTs decreased the C22:6n-3 content compared to the diet supplemented with 400 mg/kg (*p* ≤ 0.05).

**Figure 1:**
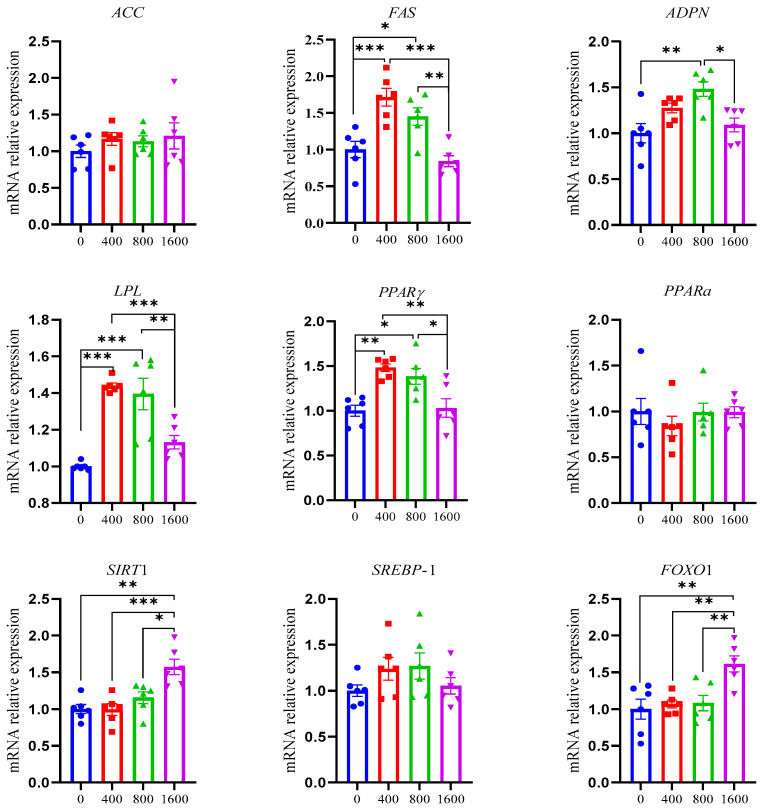
Effects of MHTs on the expression of lipid metabolism-related genes in pectoralis major muscle in Zhongshan ducks. Dietary treatments of 0, 400, 800, and 1600 were basal diets supplemented with 0, 400, 800, and 1600 mg/kg MHTs, respectively. Values are shown as the mean ± SEM. * *p* < 0.05, ** *p* < 0.01, and *** *p* < 0.001. The results are presented as means and SEMs (*n* = 6). *ACC*, acetyl-coenzyme A carboxylase; *FAS*, fatty acid synthetase; *PPARα/γ*, peroxisome proliferator-activated receptor alpha/gamma; *ADPN*, adiponectin; *SREBP*-1, sterol regulatory element-binding protein 1; *LPL*, lipoprotein lipase; *SIRT*1, sirtuin 1; *FOXO*1, forkhead transcription factor.

The lipid metabolism-related gene expression *pectoralis major* muscle in Zhongshan shelducks is shown in Figure 1. The abundance of *FAS* (fatty acid synthetase), *PPARγ* (peroxisome proliferator-activated receptor gamma), and *LPL* (lipoprotein lipase) was higher in the 400 mg/kg and 800 mg/kg MHT groups compared to that in the CON and 1600 mg/kg MHT groups (*p* ≤ 0.05) and was not significantly different between the 400 mg/kg and 800 mg/kg MHT groups or between the CON and 1600 mg/kg MHT groups (*p* > 0.05). The expression of *SIRT*1 (sirtuin 1) and *FOXO*1 (forkhead transcription factor) was highest in the 1600 mg/kg MHT group among the four groups (*p* ≤ 0.05) and was not significantly different among the CON, 400 mg/kg, and 800 mg/kg MHT groups (*p* > 0.05). The *ADPN* (adiponectin) abundance was higher in the 800 mg/kg MHT group compared to the CON and 1600 mg/kg MHT groups (*p* ≤ 0.05) and was not significantly different among the CON, 400 mg/kg, and 1600 mg/kg MHT groups (*p* > 0.05). The abundance of *ACC* (acetyl-coenzyme A carboxylase), *PPARα*, and *SREBP*-1 (sterol regulatory element-binding protein 1) was not significantly different among the four groups (*p* > 0.05).

## 4. Discussion

In this study, diets supplemented 400 and 800 mg/kg MHTs significantly improved the FBW and ADG, but 1600 mg/kg MHTs did not affect the FBW and ADG of Zhongshan shelducks, suggesting the growth-promoting effect of MHTs in Zhongshan shelducks in a dose-dependent manner. This may be attributed to the appropriate dosage of MHTs improving antioxidant capacity and nutrient digestibility and utilization by astringent effects and prolonging the retention time of feed in the small intestine to weaken the peristalsis rate of the small intestine [5,6,7], while high-dose MHTs displayed antinutritional factor effects with the dose increase [9]. Zhang et al. [18] reported that dietary supplementation with 250 and 500 mg/kg hydrolyzed tannins significantly improved the ADG, ADFI, and growth of broilers. Some studies indicate that hydrolyzed tannins do not affect animal growth and even display negative effects on growth. Tong et al. [23] revealed that diet supplemented with hydrolyzed gallic tannins (150, 300, and 450 mg/kg) had no significant effect on the growth performance of broilers. Buyse et al. [9] reported that high-dose tannins decreased broiler performance. These results suggest that the quality, dose, and shape of hydrolyzed tannins as well as the species of animals may limit their absorption and metabolization as well as result in different applicative effects of hydrolyzed tannins on animal growth. Meanwhile, hydrolyzed tannins improve animal growth by influencing the structure of intestinal microorganisms to improve the microecological environment of intestines [28]. Therefore, hydrolyzed tannins are used as feed additives for improving growth performance in livestock and poultry production. Studies suggest that hydrolysable tannins are readily hydrolyzed into polyphenol products such as gallic acids (gallotannins) and ellagic acids (ellagitannins), which display anti-inflammatory, antioxidant, and immune effects [29,30,31]. Wang et al. [32] found that dietary ellagic acid improved the ADG and decreased F/G in yellow-feathered broilers. Therefore, hydrolysable tannins promote animal growth, owing to their functional hydrolysates. Slaughter performance is an important indicator for evaluating the meat production performance of livestock and poultry. The results of this study showed that the diet supplemented with 800 mg/kg rather than 400 or 1600 mg/kg MHTs significantly increased the *pectoralis major* muscle yield of Zhongshan shelduck, which is consistent with the study of Wu et al. [33] in which tannins improved the chest muscle percentage of Luhua chickens. Dietary MHTs increased the *pectoralis major* muscle yield of Zhongshan shelducks probably by promoting protein synthesis and deposition through regulating the mTOR signaling pathway [34]. Therefore, dietary MHTs can improve growth and productivity in the Zhongshan shelduck industry.

Antioxidant capacity affects animal health, growth, and productivity [17,18]. Hydrolyzed tannins, as natural antioxidants, inhibit the production of oxidative free radicals due to a strong capacity for Fe^2+^ and remove 2,2-Diphenyl-1-picrylhydrazylradical (DPPH) and O_2_^−^·due to containing the phenolic hydroxyl group of an excellent hydrogen donor [35,36]. Previous studies discovered that diets supplemented with hydrolyzed gallotannin and ellagic acid, respectively, increased the antioxidant capacity of liver and serum in yellow-feather broilers [23,32] and hydrolysable tannins increased intestinal antioxidant capacity in weaned piglets [10]. Therefore, we infer that that diets supplemented with MHTs probably improve antioxidant capacity in Zhongshan shelducks. In this study, diets supplemented with 400 and 800 mg/kg rather than 1600 mg/kg MHTs improved antioxidant capacity by increasing GSH-Px activity and T-AOC in the *pectoralis major* muscle of Zhongshan shelducks, which is consistent with the report of Wang et al. [37] in which chestnut tannins improved muscular antioxidative capacity. Antioxidant capacity reflects animal health status, by which 400 and 800 mg/kg MHTs can promote the growth of Zhongshan shelducks. A previous study reported that increased antioxidant capacity improved meat quality in animals [1], due to which we infer that dietary MHTs might improve meat quality in Zhongshan shelducks.

Meat quality determines consumer preferences, and meat color is one of its main sensory properties [16]. Tannins might induce a reduction in meat redness owing to its binding to heme iron and reduction in iron absorption or metabolism [36,38,39,40]. The results of this study indicated that MHTs did not affect meat color compared to the CON group, suggesting that 400–1600 mg/kg MHTs might not affect iron absorption and metabolism in *pectoralis major* muscle in Zhongshan shelducks, which needs to be explored through further studies. Interestingly, high-dose MHTs improved the b* value of *pectoralis major* muscle with an increase in MHT dose compared to low-dose MHTs, probably attributable to a negative correlation between the b* value and water-holding capacity [41]. Furthermore, MHTs enhanced muscular antioxidant capacity, which might have effectively reduced the damage of lipid peroxidation to maintain muscle cell integrity and reduce water exudation from the muscle surface to increase water-holding capacity [42]. Wang et al. [37] also reported that chestnut tannins improved lamb meat quality by enhancing antioxidative capacity. In this study, 400 mg/kg and 800 mg/kg MHTs reduced drip loss at 48 h postmortem and 400 mg/kg MHTs decreased the shear force of *pectoralis major* muscle in Zhongshan shelducks, which might have resulted in the advantages of the flavor, juiciness, and nutritional status of the muscle [43]. IMF has a positive effect on muscle juiciness, tenderness, and flavor, which is regulated by lipid metabolism in the uptake of fatty acid precursors, fatty acid synthesis, fat synthesis, and fat deposition [19,20]. In this study, dietary MHTs significantly reduced the shear force and increased the content of IMF and flavor substances IMP in *pectoralis major* muscle in Zhongshan duck. Li et al. [44] reported that hydrolyzed tannins affected blood lipid levels and lipid metabolism in tissues. Therefore, we infer that dietary MHTs might affect fatty acid composition and lipid metabolism in *pectoralis major* muscle in Zhongshan ducks.

The content and composition of fatty acids in muscle affect meat quality like meat color, juiciness, flavor, and nutrition [21]. In the present study, dietary MHTs (400 mg/kg and 800 g/kg) altered fatty acid composition by reducing the content of C12:0 and C14:0 and increasing the contents of UFAs, MUFAs, C16:1n-7, C18:1n-9 (oleic acid), C18:2n-6, C20:4n-6, and C22:6n-3 (DHA) in *pectoralis major* muscle. Previous studies reported that SFAs are negatively correlated with meat flavor, while C12:0 and C14:0, among them, are recognized as the most atherogenic agents; meanwhile, UFAs are effective in preventing cardiovascular disease and MUFAs, C16:1n-7, C18:1n-9, C18:2n-6, and C20:4n-6 are positively correlated with meat flavor [45,46,47,48]. Furthermore, DHAs and oleic acid were positively associated with the nutritional and functional values of meat [49,50,51]. These results suggest that 400 and 800 mg/kg MHT supplementation improved the meat flavor, nutritional value, and functional value of Zhongshan ducks. Vlaicu et al. reported that gallic acid-rich plant additives increased UFAs and meat quality in broilers [52]. However, UFAs contain double bonds and have a loose and unstable molecular structure, which is prone to hydrogenation reactions and negatively affects the nutritional and economic values of meat products [49,50,51]. Mancini et al. [53] reported that dietary chestnut and quebracho tannins inhibited lipid peroxidation in raw meat and cooked meat. Therefore, dietary MHTs might inhibit UFA oxidation and maintain UFA stability by increasing antioxidant capacity in muscle, which might counteract the negative effect of UFAs on meat quality [54]. Ren et al. [17] found that microencapsulated Galla chinensis tannins improved antioxidant capacity and lipid metabolism in broiler chickens.

In this study, dietary MHTs (400 and 800 mg/kg) improved the transcript abundance of *FAS*, *LPL*, and *PPARγ* in *pectoralis major* muscle, which displayed a similar trend to IMF content in Zhongshan shelducks. PPARγ is involved in fatty acid synthesis and transport and induces adipocyte differentiation, lipid metabolism, and IMF deposition in muscle by activating the PPAR signaling pathway [55,56]. FAS regulates the de novo synthesis of long-chain fatty acids and promotes fat deposition [57]. LPL, as a key speed-limiting enzyme in triglyceride hydrolysis, promotes free fatty acid uptake in adipocytes and myocytes in muscles and IMF deposition [58]. The activation of PPARγ promotes ADPN signaling to alleviate lipid accumulation in adipose [59] but increases the expression of FAS [60] and LPL [61] to improve lipid synthesis and fatty acid uptake. This suggests that 400 and 800 mg/kg MHTs stimulate fatty acid uptake, lipid synthesis, and IMF deposition by activating *PPARγ* to enhance the expression of *FAS* and *LPL* [60,61]. In this study, the diet supplemented with 1600 mg/kg MHTs decreased the expressions of *SIRT*1, *FOXO*1, and *PPAR*γ in *pectoralis major* muscle in Zhongshan duck, indicating that high-dose MHTs might inhibit adipogenesis and deposition and promote fat catabolism by activating *SIRT*1 to up-regulate *FOXO*1 and down-regulate *PPARγ* [62,63]. Ren et al. [17] reported that microencapsulated Galla chinensis tannins (300 mg/kg) improved hepatic lipid accumulation by activating the SIRT1/SREBP-1 pathway and improved fatty acid β-oxidation. Nobushi et al. [64] reported that hydrolysable tannins inhibited lipid accumulation in adipocytes. The inconsistencies of tannins in lipid metabolism might be attributed to the differences in hydrolyzed tannin sources, animal species, and tissues.

## 5. Conclusions

Dietary supplementation with appropriate doses of MHTs can significantly improve growth performance, meat quality, and antioxidant capacity. Furthermore, MHTs dose-dependently improve IMF deposition, fatty acid profiles, and lipid metabolism by activating the *PPARγ* pathway to regulate the expression of *FAS* and *LPL*. The present study demonstrated that diets supplemented with low-dose MHTs (400–800 mg/kg) displayed positive effects on growth or meat quality in Zhongshan shelducks. These findings provide an effectively applicative strategy and theoretical reference for MHTs for meat quality attributes in the poultry industry. However, further studies are needed to determine antioxidant pathways and the relationship between lipid metabolism pathways and antioxidant regulation pathways in Zhongshan shelducks supplemented with MHTs. Further, the effects of MHTs on iron absorption in Zhongshan shelducks still need to be confirmed.

## Figures and Tables

**Table 1:** Composition and nutrient levels of diets (as-fed basis, %).

Ingredients	Content
Corn	58.00
Soybean meal	12.10
Wheat middlings	23.70
Soybean oil	2.00
L-Lys·HCl	0.10
NaCl	0.30
CaHPO4	1.00
Limestone	1.80
Premix	1.00
Total	100.00
Nutrient level	
Crude protein	14.00
Calcium	1.19
Total phosphorus	0.68
Lysine	0.75
Methionine	0.25
Threonine	0.51
Tryptophan	0.17
Methionine + Cystine	0.75
ME (MJ/kg)	13.06

The premix provided the following per kilogram of diet: vitamin A, 9875 IU; vitamin D3, 2500 IU; vitamin E, 13.1 IU; vitamin K3, 2.3 IU; nicotinic acid, 37.5 mg; pantothenic acid, 6.1 mg; pyridoxine, 0.6 mg; riboflavin, 4.5 mg; biotin, 0.02 mg; folic acid, 0.5 mg; thiamine, 0.6 mg; cobalamine, 0.01 mg; Cu, 15 mg; Fe, 50 mg; Mn, 80 mg; Zn, 80 mg; Se, 0.25 mg; I, 2 mg.

**Table 2:** Lipid metabolism-related gene primer sequences.

Genes	Primer Sequence 5′→3′	GenBank Accession No.
*GAPDH*	Forward: TTGGCATTGTGGAGGGTCTT	XM_032204998.1
	Reverse: GGGTAGGGCTCTCTCCTGAT	
*ACC*	Forward: CTTCTGGACCGGTAAAGGCA	JQ080306.1
	Reverse: GACATGCTGGGCCTCCAAT	
*FAS*	Forward: GCCTCGTGTTTACCAACATGG	AY613443.1
	Reverse: ATTAAGACAGCCACGAGGAGC	
*PPARγ*	Forward: GCCCAGATGCAGATACCCTG	EF546801.2
	Reverse: AAGGGTGAAGGTGTGCTACG	
*ADPN*	Forward: CAACTGGATGGGAGGAGCAC	DQ452618.1
	Reverse: GTACTGAGCTCAACACCCGT	
*SREBP*-1	Forward: AGCAACCAGAAGCTGAAGCA	JQ080310.1
	Reverse: AGGGACTTGCTCTTCTGCAC	
*LPL*	Forward: CATGGATGGACGGTGACAGG	FJ859348.1
	Reverse: CCACCAGCTTAGTGTACGCA	
*SIRT*1	Forward: TCGCGCCATGAAGTATGACA	XM_032191260.1
	Reverse: CATGGGGGATGGAACTTGGA	
*FoxO*1	Forward: GGATGTAGTAGGAGGCTACGGT	XM_005014856.5
	Reverse: ACTCTGTCTGCTGCCAACTC	
*PPARa*	Forward: GCTTGTGAAGGTTGTAAGGGTT	NM_001310383.1
	Reverse: CATTCCAACTGAAAGGCACT	

*ACC*: acetyl-CoA carboxylase; *FAS*: fatty acid synthase; *PPARγ/a*: peroxisome proliferator-activated receptor gamma/alpha; *ADPN*: adiponectin; *SREBP*-1: sterol regulatory element-binding protein 1; *LPL*: lipoprotein lipase; *SIRT*1: sirtuin 1; *FoxO*1: forkhead box O1.

**Table 3:** Effect of MHTs on growth and slaughter performance of Zhongshan shelducks.

Items	MHT Supplementation, mg kg^−1^				SEM	*p*-Value
	0	400	800	1600		
IBW, g	1790.24	1790.85	1790.31	1790.65	0.14	0.819
FBW, g	2253.50 ^c^	2402.75 ^ab^	2463.69 ^a^	2332.81 ^bc^	45.29	0.001
ADG, g	7.72 ^c^	10.20 ^ab^	11.22 ^a^	9.04 ^bc^	0.75	0.001
Dressing percentage, %	80.43	82.28	83.13	82.18	0.57	0.908
Semi-eviscerated yield, %	66.92	74.48	75.06	72.25	1.85	0.165
Eviscerated yield, %	62.37	66.34	67.55	67.90	1.27	0.453
*Pectoralis major* muscle yield, %	9.02 ^b^	9.63 ^b^	11.12 ^a^	9.62 ^b^	0.45	0.002
Thigh muscle yield, %	6.60	7.13	7.38	7.62	0.22	0.432

^a,b,c^ Means in a row without a common superscript differ at *p* ≤ 0.05. The results are presented as means and SEMs (*n* = 6). MHTs: microencapsulated hydrolyzed tannins; SEM: standard error of mean; IBW: initial body weight; FBW: final body weight; ADG, average daily gain.

**Table 4:** Effect of MHTs on antioxidant parameters in *pectoralis major* muscle of Zhongshan shelducks.

Items	MHT Supplementation, mg kg^−1^				SEM	*p*-Value
	0	400	800	1600		
GSH-Px (U/mgprot)	43.74 ^b^	48.72 ^a^	47.67 ^a^	45.59 ^ab^	1.11	0.045
T-SOD (U/mgprot)	28.39	31.35	32.00	27.19	1.16	0.218
MDA (nmol/mgprot)	0.30	0.27	0.28	0.26	0.01	0.276
T-AOC (U/mgprot)	2.16 ^b^	2.66 ^a^	2.51 ^a^	2.14 ^b^	0.13	0.004

^a,b^ Means in a row without a common superscript differ at *p* ≤ 0.05. The results are presented as means and SEMs (*n* = 6). MHTs: microencapsulated hydrolyzed tannins; SEM: standard error of mean; GSH-Px: glutathione peroxidase; SOD: total superoxide dismutase; MDA: malondialdehyde; T-AOC: total antioxidant capacity.

**Table 5:** Effect of MHTs on meat quality of *pectoralis major* muscle in Zhongshan shelducks.

Items	MHT Supplementation, mg kg^−1^				SEM	*p*-Value
	0	400	800	1600		
pH_45 min_	5.93	5.91	6.09	5.96	0.04	0.534
L*_45 min_	33.30	34.13	33.97	36.61	0.73	0.294
a*_45 min_	20.50	20.20	21.23	21.26	0.27	0.868
b*_45 min_	1.74 ^ab^	1.45 ^b^	2.03 ^a^	2.09 ^a^	0.15	0.004
pH_24 h_	5.89	5.92	5.96	5.89	0.02	0.502
L*_24 h_	34.29	33.70	35.65	35.86	0.52	0.572
a*_24 h_	20.26	20.15	20.10	20.81	0.16	0.808
b*_24 h_	1.06	1.24	1.29	1.20	0.05	0.921
pH_48 h_	5.93	5.87	6.01	5.93	0.03	0.243
L*_48 h_	33.97	33.60	35.53	35.86	0.56	0.596
a*_48 h_	22.24	21.21	22.03	22.47	0.27	0.645
b*_48 h_	1.85	1.50	2.10	1.77	0.12	0.071
Drip loss _24 h_	2.12	1.98	1.93	2.22	0.07	0.441
Drip loss _48 h_	2.54 ^ab^	2.24 ^b^	2.46 ^b^	2.67 ^a^	0.09	0.002
Shear force, N	59.95 ^a^	52.88 ^b^	58.67 ^ab^	63.41 ^a^	2.19	0.016
IMP (%)	0.51 ^b^	0.81 ^a^	0.89 ^a^	1.10 ^a^	0.12	<0.001
IMF (%)	4.53 ^b^	5.62 ^a^	5.55 ^a^	4.57 ^b^	0.30	0.001

^a,b^ Means in a row without a common superscript differ at *p* ≤ 0.05. The results are presented as means and SEMs (*n* = 6). MHTs: microencapsulated hydrolyzed tannins; SEM: standard error of mean; IMP: inosine monophosphate; IMF: intramuscular fat.

**Table 6:** Effect of MHTs on fatty acid content of *pectoralis major* muscle in Zhongshan ducks (mg/100 g meat).

Items	MHT Supplementation, mg kg^−1^				SEM	*p*-Value
	0	400	800	1600		
C11:0 (Hendecanoic Acid)	59.67	59.10	59.83	60.63	0.32	0.106
C12:0 (Lauric Acid)	66.60 ^a^	40.63 ^c^	59.87 ^ab^	47.80 ^bc^	5.85	<0.001
C14:0 (Myristic Acid)	52.20 ^a^	30.73 ^c^	43.83 ^b^	38.80 ^b^	4.50	0.004
C16:0 (Palmitic Acid)	346.97	286.23	301.03	275.70	15.71	0.236
C18:0 (Stearic Acid)	106.33	97.13	98.77	84.23	4.64	0.054
C16:1n-7 (Palmitoleic Acid)	29.67 ^b^	34.93 ^a^	26.30 ^bc^	24.00 ^c^	2.37	<0.001
C18:1n-9 (Oleic Acid)	287.40 ^c^	394.07 ^a^	341.3 ^b^	290.47 ^c^	25.16	<0.001
C20:1n-9 (Eicosenoic Acid)	4.30	4.17	4.43	4.33	0.06	0.859
C18:2n-6 (Linoleic Acid)	252.50 ^cd^	314.83 ^a^	297.53 ^ab^	272.47 ^bc^	13.72	<0.001
C18:3n-3 (Linolenic Acid)	14.30	16.67	13.77	14.87	0.63	0.357
C20:2n-6 (Eicosadienoic Acid)	3.87 ^a^	3.50 ^b^	3.87 ^a^	3.70 ^ab^	0.09	0.0002
C20:4n-6 (Arachidonic Acid)	100.00 ^b^	118.00 ^a^	101.70 ^b^	92.30 ^bc^	5.40	0.003
C22:6n-3 (Docosahexaenoic Acid, DHA)	6.27 ^ab^	8.17 ^a^	5.77 ^b^	5.13 ^b^	0.65	0.0049
SFA	632.10	513.83	563.40	507.10	28.87	0.0597
MUFA	321.40 ^c^	433.17 ^a^	372.13 ^b^	318.80 ^c^	26.90	<0.001
PUFA	377.002 ^c^	461.13 ^a^	422.70 ^ab^	388.50 ^bc^	18.94	<0.001
UFA	698.40 ^c^	894.30 ^a^	794.80 ^b^	707.30 ^c^	45.71	<0.001

^a,b,c,d^ Means in a row without a common superscript differ at *p* ≤ 0.05. The results are presented as means and SEMs (*n* = 6). MHTs: microencapsulated hydrolyzed tannins; SEM: standard error of mean; SFAs: saturated fatty acids = C11:0 + C12:0 + C14:0 + C16:0 + C18:0; MUFAs: monounsaturated fatty acids = C16:1n-7 + C18:1n-9 + C20:1n-9; PUFAs: polyunsaturated fatty acids = C18:2n-6 + C18:3n-3 + C20:2n-6 + C20:4n-6 + C22:6n-3; UFAs: unsaturated fatty acids = C16:1n-7 + C18:1n-9 + C20:1n-9 + C18:2n-6 + C18:3n-3 + C20:2n-6 + C20:4n-6 + C22:6n-3.

## Data Availability

The original contributions presented in this study are included in the article. Further inquiries can be directed to the corresponding authors.

## Abbreviations

The following abbreviations are used in this manuscript:

MHTs	Microencapsulated hydrolyzed tannins
IBW	Initial body weight
FBW	Final body weight
ADG	Average daily gain
T-AOC	Total superoxide dismutase
T-SOD	Total superoxide dismutase
GSH-Px	Glutathione peroxidase
MDA	Malondialdehyde
ACC	Acetyl-CoA carboxylase
IMP	Inosine monophosphate
IMF	Intramuscular fat
SFA	Saturated fatty acid
MUFA	Monounsaturated fatty acid
PUFA	Polyunsaturated fatty acid
DHA	Docosahexaenoic acid
*FAS*	Fatty acid synthase
*PPARγ/a*	Peroxisome proliferator-activated receptor gamma/alpha
*ADPN*	Adiponectin
*SREBP*-1	Sterol regulatory element-binding protein 1
*LPL*	Lipoprotein lipasE
*SIRT*1	Sirtuin 1
*FoxO*1	Forkhead box O1
